# An integrated biochemical system for nitrate assimilation and nitric oxide detoxification in *Bradyrhizobium japonicum*

**DOI:** 10.1042/BJ20150880

**Published:** 2016-01-25

**Authors:** Juan J. Cabrera, Ana Salas, María J. Torres, Eulogio J. Bedmar, David J. Richardson, Andrew J. Gates, María J. Delgado

**Affiliations:** * Estación Experimental del Zaidín, CSIC, PO Box 419, Granada 18080, Spain; † Centre for Molecular and Structural Biochemistry, University of East Anglia, Norwich Research Park, Norwich NR4 7TJ, U.K.; ‡ School of Biological Sciences, University of East Anglia, Norwich Research Park, Norwich NR4 7TJ, U.K.

**Keywords:** bacterial denitrification, bacterial haemoglobin, nitrate reduction, nitric oxide reductase, nitrite reduction

## Abstract

Rhizobia are recognized to establish N_2_-fixing symbiotic interactions with legume plants. *Bradyrhizobium japonicum*, the symbiont of soybeans, can denitrify and grow under free-living conditions with nitrate (NO_3_^−^) or nitrite (NO_2_^−^) as sole nitrogen source. Unlike related bacteria that assimilate NO_3_^−^, genes encoding the assimilatory NO_3_^−^ reductase (*nasC*) and NO_2_^−^ reductase (*nirA*) in *B. japonicum* are located at distinct chromosomal loci. The *nasC* gene is located with genes encoding an ABC-type NO_3_^−^ transporter, a major facilitator family NO_3_^−^/NO_2_^−^ transporter (NarK), flavoprotein (Flp) and single-domain haemoglobin (termed Bjgb). However, *nirA* clusters with genes for a NO_3_^−^/NO_2_^−^-responsive regulator (NasS-NasT). In the present study, we demonstrate NasC and NirA are both key for NO_3_^−^ assimilation and that growth with NO_3_^−^, but not NO_2_^−^ requires *flp*, implying Flp may function as electron donor to NasC. In addition, *bjgb* and *flp* encode a nitric oxide (NO) detoxification system that functions to mitigate cytotoxic NO formed as a by-product of NO_3_^−^ assimilation. Additional experiments reveal NasT is required for NO_3_^−^-responsive expression of the *narK-bjgb-flp-nasC* transcriptional unit and the *nirA* gene and that NasS is also involved in the regulatory control of this novel bipartite assimilatory NO_3_^−^/NO_2_^−^ reductase pathway.

## Abstract (precis)

We report a dual functional system for bacterial nitrate (NO_3_^−^) assimilation and nitric oxide (NO) detoxification. The assimilatory NO_3_^−^ reductase (NasC) can generate nitric oxide (NO). Co-expression of an NO-detoxification system acts to counteract accumulation of cytotoxic NO during anaerobic NO_3_^−^-dependent growth.

## INTRODUCTION

Fixation of atmospheric dinitrogen (N_2_) by plant-associated symbiotic soil bacteria, collectively termed rhizobia, is a significant agricultural process that reduces dependence on synthetic nitrogen (N) containing fertilizers in crop production. This protects water quality and human health as well as the wider environment. In addition to N_2_ fixation, the soybean endosymbiont *Bradyrhizobium japonicum* USDA110 is capable of growing anaerobically with the water-soluble nitrate (NO_3_^−^) anion, as an alternative terminal electron acceptor to oxygen (O_2_), which is reduced to N_2_ gas by respiratory denitrification. During this process, several free N-containing intermediates are produced, including: (i) the oxyanion nitrite (NO_2_^−^), (ii) the gaseous cytotoxic free-radical nitric oxide (NO) and (iii) the potent and long-lived greenhouse gas nitrous oxide (N_2_O). In *B. japonicum*, the denitrification apparatus is encoded by the *napEDABC*, *nirK*, *norCBQD* and *nosRZDFYLX* genes, which express the periplasmic NO_3_^−^ reductase (NapABC), copper-containing NO_2_^−^ reductase (NirK), cytochrome-*c* NO reductase (NorCB) and N_2_O reductase (NosZ) enzymes, respectively [1]. This bacterium is distinguished by the ability to denitrify under both free-living and symbiotic conditions [2–4].

Several reports suggest that rhizobial denitrification is the main driver for production and release of the environmentally damaging agents NO and N_2_O from alfalfa and soybean nodules [5–8]. NO is a highly reactive and well-studied ozone-depleting agent, whereas N_2_O is increasingly recognized as a powerful greenhouse gas with an estimated 300-fold higher radiative potential for global warming, molecule for molecule, compared with carbon dioxide [9–11]. Importantly, in active root nodules, NO also acts as a potent inhibitor of nitrogenase, the central enzyme of symbiotic N_2_-fixation [8,12]. Under free-living denitrifying conditions, the *B. japonicum* proteins NirK and NorCB are physiologically important for the synthesis and detoxification of NO, respectively [1]. However, several studies suggest the involvement of other sinks for NO that are distinct from the recognized denitrification pathway in nodules [8,13]. For example, in related bacteria, NO may be oxidized to NO_3_^−^ or reduced to N_2_O by cytoplasmic detoxification enzymes. These systems include single-domain haemoglobins (sdHbs), truncated haemoglobins (trHbs), flavohaemoglobins (FHbs) and flavorubredoxin (FlRd) [14–18].

Following sequencing of the *B. japonicum* USDA110 genome [19], several studies have investigated the involvement of a putative bacterial sdHb, termed Bjgb, in NO-detoxification, under free-living conditions [3,20]. This bacterial haemoglobin is encoded by the ORF blr2807 and resides within a cluster of other uncharacterized ORFs (blr2803–09) predicted to encode components of a NO_3_^−^ assimilation (Nas) pathway, including: an ABC-type NO_3_^−^ transport system (blr2803–05), a major facilitator superfamily (MFS)-type NO_3_^−^/NO_2_^−^ transporter (blr2806), an FAD-dependent NAD(P)H oxidoreductase (blr2808) and the catalytic subunit of the assimilatory NO_3_^−^ reductase (blr2809), termed NasC (we note the gene for the assimilatory NO_3_^−^ reductase in *B. japonicum* was previously termed *nasA*, but here we unify the gene nomenclature for α-proteobacteria). The genome also contains a putative ferredoxin-dependent assimilatory NO_2_^−^ reductase (NirA) that is present at bll4571, a distinct locus on the chromosome. This putative *nirA* gene lies immediately downstream of genes recently reported to code for a NO_3_^−^/NO_2_^−^ responsive regulatory system (NasS-NasT), similar to that characterized in the model NO_3_^−^-utilizing soil bacterium *Paracoccus denitrificans* PD1222 [21,22]. However, to date, a role for the proteins encoded at blr2803–09 and bll4571–73 loci in NO_3_^−^/NO_2_^−^ assimilation and conceivably NO management in *B. japonicum* remains to be established.

Although the biochemical components for Nas systems may be highly modular, in related α-proteobacteria such as *P. denitrificans* and *Rhodobacter capsulatus* E1F1, genes encoding regulatory and structural elements for the NO_3_^−^ assimilation pathway are typically found together [23,24]. For example, in *P. denitrificans*, the genes required for import and reduction of NO_3_^−^ and/or NO_2_^−^ are encoded by *nasABGHC* and the *nasTS* genes required for NO_3_^−^/NO_2_^−^-responsive regulatory control are found immediately upstream [25]. Here, the assimilatory NO_3_^−^/NO_2_^−^ reductase apparatus includes a: NO_3_^−^/NO_2_^−^ transporter (NasA), NO_2_^−^ reductase (NasB), ferredoxin (NasG), NO_2_^−^ transporter (NasH) and NO_3_^−^ reductase (NasC). Notably, the *nasG* gene is highly conserved in NO_3_^−^/NO_2_^−^ assimilation gene clusters, which is consistent with a key role for the NasG ferredoxin in mediating electron flux from the NADH-oxidizing site in NasB to the sites of NO_3_^−^ and NO_2_^−^ reduction in NasC and NasB respectively, in order to prevent intracellular accumulation of NO_2_^−^ [25]. In *P. denitrificans*, the RNA-binding protein NasT has been recently shown to positively and directly regulate *nas* expression (i.e. *nasABGHC*) by interacting with the *nasA*-leader mRNA. The NO_3_^−^/NO_2_^−^-binding sensor NasS controls NasT activity and the NasS and NasT proteins co-purify as a stable heterotetrameric regulatory complex, NasS-NasT in the absence of inducer [21]. The NasS-NasT system from *B. japonicum* has now been characterized by Sánchez et al. [22] and shown to regulate expression of *napE* and *nosZ* genes for the dissimilatory denitrification pathway.

The processes and enzymes for rhizobial denitrification have been well studied [1–4]; however, the biochemical apparatus for NO_3_^−^/NO_2_^−^ assimilation by plant-associated rhizobia has yet to be characterized. In the present work, we demonstrate a dual role for the blr2806–09 and bll4571–73 loci for NO_3_^−^/NO_2_^−^ assimilation and NO management in *B. japonicum*.

## EXPERIMENTAL

### Bacterial strains, plasmids and growth conditions

The bacterial strains and plasmids used in the present study are listed in Supplementary Table S1. Gene deletion and transcriptional reporter construction used previously established methods [26,27], with key modifications as outlined below. *B. japonicum* strains were grown routinely under aerobic conditions at 30°C in peptone-salts-yeast extract (PSY) medium supplemented with 0.1% (w/v) L-arabinose [28]. Growth curves for different N-sources were performed in Bergersen minimal medium [29] supplemented with 10 mM KNO_3_ (BN3), 1 mM NaNO_2_ (BN2) or 10 mM KNO_3_ plus 6.5 mM L-glutamate (BGN3) as sole N-sources and incubated aerobically or anaerobically. Anaerobic conditions were reached by incubating the cells in completely filled glass serum tubes. Growth was followed by measuring attenuance (*D*) of cell cultures at 600 nm.

To test growth inhibition by nitrosative stress, cells were grown in Bergersen minimal medium with 6.5 mM L-glutamate (BG) as sole N-source and incubated under microaerobic conditions in serum tubes sealed with rubber septa. The headspace of these tubes was filled with a gas mixture of 2% (v/v) O_2_ and 98% (v/v) N_2_ and was replaced with fresh gas mixture every 24 h. Nitrosative stress was induced by adding 1 mM sodium nitroprusside (SNP) or spermine NONOate to the cultures 24 h after inoculation. To test cell survival after nitrosative stress induction, cells were grown to early stationary growth phase under the same conditions as for growth inhibition experiments (final *D* value at 600 nm was ∼0.5). Then, 1 mM SNP or spermine NONOate was added to the cultures (replica cultures were not subjected to nitrosative stress as controls). Cell cultures were incubated at 30°C and 0.1 ml of samples were taken periodically, serially diluted in growth medium and plated into PSY-agar. Colonies were counted after incubation for 7 days under aerobic conditions. The capacity for colony formation of control cells was considered as 100% survival.

To induce the expression of NorCB as a cellular marker for NO, *B. japonicum* was grown in Bergersen minimal medium where glycerol was replaced with 10 mM succinate as carbon source (BS) [30,31]. The medium was supplemented with 10 mM KNO_3_ as sole N-source (BSN3). In these experiments, the headspace was filled with 2% (v/v) O_2_ and 98% N_2_ (v/v). In contrast with microaerobic growth conditions, the atmosphere for cultures was not replaced. As such, cells consumed the O_2_ present and reached anoxic conditions after 24 h incubation.

Antibiotics were added to *B. japonicum* cultures at the following concentrations (μg·ml^−1^): chloramphenicol 20, tetracycline 100, spectinomycin 200, streptomycin 200 and kanamycin 200. *Escherichia coli* strains were cultured in LB medium [32] at 37°C including tetracycline 10, spectinomycin 20, streptomycin 20, kanamycin 30 and ampicillin at 200 μg·ml^−1^. *E. coli* S17-1 served as the donor for conjugative plasmid transfer [33].

### Construction of *B. japonicum narK-lacZ* and *nirA-lacZ* transcriptional fusions

For construction of transcriptional fusion reporter plasmids, DNA fragments for the *narK* (520 bp) and *nirA* (563 bp) promoter regions were amplified using primers *narK-lacZ*_For/*narK-lacZ*_Rev and *nirA-lacZ*_For/*nirA-lacZ*_Rev, respectively (see Supplementary Table S2 for oligonucleotide sequences). Fragments were digested with PstI or PstI-EcoRI and cloned into the *lacZ* fusion vector pSUP3535, which is derived from pSUP202 [33] to give plasmids pDB4009 and pDB4018 respectively (Supplementary Table S1). The correct orientation of cloned inserts was verified by sequencing.

Transcriptional fusion plasmids pDB4009 and pDB4018 were integrated by homologous recombination into the chromosome of wild-type (WT) *B. japonicum* USDA110 and *nasS* and *nasT* mutants to produce strains 4009, 4012-4009, 4013-4009, 4018, 4012-4018 and 4013-4018 detailed in Supplementary Table S1. Correct recombination was checked by PCR analysis of genomic DNA isolated from each strain.

### Growth conditions for *β*-galactosidase activity assay of *narK*-*lacZ*, *nirA*-*lacZ* and *norC*-*lacZ* fusions

Strains 4009, 4012-4009, 4013-4009, 4018, 4012-4018 and 4013-4018 containing the *narK*-*lacZ* or *nirA*-*lacZ* reporter-fusion constructs (Supplementary Table S1) were grown aerobically in PSY medium. Cells were harvested by centrifugation at 8000 ***g*** for 10 min, washed twice with nitrogen-free Bergersen medium and cultured aerobically in the same medium or in BN3 medium, for 48 h (until a *D* value of ∼0.5 at 600 nm was obtained). To measure *β*-galactosidase activity from the *norC*-*lacZ* fusion, plasmid pRJ2499 (Supplementary Table S1) was integrated by homologous recombination into the chromosome of *bjgb*, *nasC*, *napA* and *nasC*, *napA* mutants to produce strains 4001-2499, 4003-2499, GRPA1-2499 and GRPA1-4003-2499 respectively (Supplementary Table S1). In order to induce expression of *nor* genes, cells were cultured in BSN3 medium with 2% (v/v) initial O_2_ concentration.

### Construction and complementation of *B. japonicum* mutants

Genomic and plasmid DNA isolation was carried out using the REALPURE Genomic DNA purification Kit (Real) and Qiagen Plasmid Kit (Qiagen) respectively. Custom oligonucleotide primers were supplied by Sigma, PCR was performed using the High Fidelity DNA polymerase Phusion enzyme (Thermo) and DNA digestions were carried out using Fast digest enzymes (Fermentas). All mutants constructed in the present work were made by in frame deletion of the corresponding gene using the mobilizable pK18*mobsacB* suicide vector that conferred kanamycin resistance and sucrose sensitivity on the host (Supplementary Table S1). To generate mutant strains, upstream and downstream regions of relevant target genes were amplified by PCR using the gene-specific primer sets detailed in Supplementary Table S2.

For the *narK* deletion mutant, upstream (834 bp) and downstream (981 bp) DNA fragments flanking blr2806 were amplified by PCR using blr2806_up_For/ blr2806_up_Rev and blr2806_down_For/blr2806_down_Rev primer pairs (Supplementary Table S2). The 981-bp fragment was inserted into pK18*mobsacB* as an XbaI-HindIII fragment that contained a new unique XhoI restriction site immediately downstream of the XbaI site. Subsequently, the 834-bp fragment was inserted into this plasmid as a BamHI-XhoI fragment yielding plasmid pDB4000. Double recombination of pDB4000 with the *B. japonicum* genome led to the replacement of the WT blr2806 gene encoding a 459 amino acid (aa) protein for an in frame truncated version encoding 38 aa.

To generate *bjgb* and *nasT* deletion mutants, upstream and downstream regions flanking blr2807 (824 and 884 bp fragments) and bll4573 (696 and 736 bp fragments) were amplified by PCR using gene-specific primer pairs, i.e. blr2807_up_For/blr2807_up_Rev and blr2807_down_For/blr2807_down_Rev for blr2807 and bll4573_up_For/bll4573_up_Rev and bll4573_down_For/bll4573_down_Rev for bll4573 (Supplementary Table S2). The PCR products containing the upstream regions of blr2807 and bll4573 were inserted separately into pK18*mobsacB* as EcoRI-XbaI fragments and subsequently the downstream PCR products were inserted into the respective plasmids as XbaI-HindIII DNA fragments yielding plasmids pDB4001 and pDB4013 (Supplementary Table S1). Double recombination of pDB4001 and pDB4013 with the *B. japonicum* genome led to the replacement of the blr2807 gene encoding a 142 aa protein and the bll4573 gene encoding a 196 aa protein with in-frame truncated versions of 33- and 27-aa for blr2087 or bll4573, respectively.

To generate the *flp* and *nasC* mutants, upstream and down-stream regions of blr2808 (983 and 856 bp) and blr2809 (829 and 840 bp) were amplified by PCR using gene-specific primer pairs blr2808_up_For/blr2808_up_Rev and blr2808_down_For/blr2808_down_Rev for blr2808 and bll2809_up_For/bll2809_up_Rev and bll2809_down_For/bll2809_down_Rev for bll2809 (Supplementary Table S2). The PCR product containing the upstream regions of blr2808 and blr2809 were cloned into separate pK18*mobsacB* plasmids as EcoRI-BamHI fragments and subsequently the downstream DNA regions were inserted into the relevant plasmid as BamHI-HindIII fragments to give pDB4002 and pDB4003. Double recombination of either pDB4002 or pDB4003 with the *B. japonicum* genome led to the replacement of the blr2808 gene (encoding a 418-aa protein) or the blr2809 gene (encoding a 901-aa protein) for in-frame truncated versions encoding a 37- or 27-aa peptide respectively.

The *ntrABC* mutant was generated by double recombination of *B. japonicum* USDA110 genomic DNA with plasmid pDB4004 (Supplementary Table S1). To generate pDB4004, the upstream region of blr2803 (773 bp) and the downstream region of blr2805 (721 bp) were amplified by PCR using primer pairs blr2803_up_For/blr2803_up_Rev and blr2805_down_For/blr2805_down_Rev (Supplementary Table S2). Firstly, the PCR product containing the upstream region was digested with BamHI and XbaI restriction enzymes and cloned into pK18*mobsacB*. Then, the PCR product corresponding to the downstream region was inserted into the plasmid as an XbaI-PstI fragment to give plasmid pBD4004 (Supplementary Table S1). Double recombination of pBD4004 with the *B. japonicum* genome replaced blr2803–05 genes for an in-frame truncated version encoding a 31-aa peptide.

The *nirA* and *nasS* mutants were generated by double recombination of *B. japonicum* USDA110 genomic DNA with plasmids pDB4011 and pDB4012 respectively. To generate pDB4011 and pDB4012, the upstream and downstream regions of bll4571 (641 and 692 bp) and bll4572 (687 and 664 bp) were amplified by PCR using the primer pairs bll4571_up_For/bll4571_up_Rev and bll4571_down_For/bll4571_down_Rev for bll4571 and bll4572_up_For/bll4572_up_Rev and bll4572_down_For/bll4572_down_Rev for bll4572 (Supplementary Table S2). PCR products containing the upstream regions were cloned first into pK18*mobsacB* as EcoRI-KpnI DNA fragments. Then, amplified downstream regions were inserted into the plasmid as KpnI-XbaI fragments yielding plasmids pDB4011 and pDB4012 (Supplementary Table S1). Finally, double recombination replaced bll4571 and bll4572 genes (encoding 625- and 388-aa proteins respectively) for in-frame truncated versions encoding 17- and 23-aa peptides respectively.

All plasmids constructed for mutagenesis were sequenced and transferred via conjugation into *B. japonicum* USDA110 using *E. coli* S17-1 as donor strain. Double recombination events were favoured by first selecting single recombinants for kanamycin resistance and screening candidates by PCR. Then, selected clones containing the plasmid co-integrated in the genome were grown in PSY-agar medium supplemented with sucrose 10% (w/v) to select for double recombinants. Sucrose resistant colonies were checked for kanamycin sensitivity. Double recombinants were confirmed by PCR.

*B. japonicum* GRC131-4001 containing a double mutation in *bjgb* and *norC* was generated by transferring plasmid pJNOR43M2 [34] via conjugation into the *B. japonicum bjgb* mutant (Supplementary Table S1). Double recombinants were selected for kanamycin resistance and tetracycline sensitivity. The correct replacement of the WT *norC* gene by kanamycin resistance gene (*aphII*) insertion was checked by PCR.

*B. japonicum* GRPA1-4003 containing a double mutation in *napA* and *nasC* was generated by transferring plasmid pBG602Ω [26] via conjugation into the *B. japonicum nasC* mutant (Supplementary Table S1) using *E. coli* S17-1 as donor strain. Double recombination events were favoured by growth on agar plates containing sucrose. Mutant strains resistant to spectinomycin/streptomycin, but sensitive to kanamycin were checked by PCR for correct replacement of the WT fragment by the Ω interposon.

The *bjgb*, *flp* and *nasC* strains were complemented with pDB4014, pDB4015, pDB4017 expression constructs containing the corresponding intact genes (Supplementary Table S1). For this, *bjgb*, *flp* and *nasC* genes were amplified by PCR using primer sets blr2807_For/blr2707_Rev, blr2808_For/blr2808_Rev and blr2809_For/blr2809_Rev (Supplementary Table S2). DNA fragments containing the relevant ORF and Shine–Dalgarno sequence were cloned separately into pTE3 vector [35]. All complementation constructs were sequenced and transferred via conjugation into the relevant *B. japonicum* mutant using *E. coli* S17-1 as donor strain. Complemented strains 4001-pDB4014, 4002-pDB4015 and 4003-pDB4017 (Supplementary Table S1) were confirmed by plasmid extraction and checked by restriction analyses and PCR.

### Analysis of gene expression by RT-PCR

Total RNA was isolated from *B. japonicum* cells grown anaerobically to a *D* of ∼0.4 (at 600 nm) in BN3 medium, as previously described [36]. First strand cDNA synthesis was performed with the SuperScript II reverse transcriptase (Invitrogen) according to the supplier's guidelines, using 1 μg of total RNA and primer e (Supplementary Table S2). cDNA generated was used for amplification of putative intergenic regions between blr2805 and blr2809, using primers pairs a1/a2 to d1/d2 (Supplementary Table S2), essentially as described by Sambrook and Russell [32]. In negative controls, reverse transcriptase was omitted and for positive controls PCR was performed with *B. japonicum* USDA110 genomic DNA as template.

### β-Galactosidase assays

β-Galactosidase activity was determined using permeabilized cells from at least three independently grown cultures assayed in triplicate for each strain and condition, as previously described [37]. The absorbance data at 420 and 600 nm were determined for all samples and cultures in a plate reader (SUNRISE Absorbance Reader, TECAN), using the software XFluor4 (TECAN) and specific activities were calculated in Miller units.

### Determination of NO_3_^−^ reductase and NO_2_^−^ reductase activity

*B. japonicum* was grown under aerobic conditions in PSY medium, harvested by centrifugation at 8000 ***g*** for 10 min at 4°C, washed twice with BN3 medium and inoculated to a *D* value of ∼0.4 (at 600 nm) in fresh minimal medium of the same composition. Following 72 h incubation under relevant conditions, cells were harvested, washed with 50 mM Tris/HCl buffer (pH 7.5) to remove excess NO_2_^−^ and then resuspended in 1 ml of the same buffer prior to assay for enzymatic activity. Methyl viologen-dependent NO_3_^−^ reductase (MV-NR) and NO_2_^−^ reductase (MV-NIR) activity was measured essentially as described by Delgado et al. [26]. The reaction mixture contained 50 mM Tris/HCl buffer (pH 7.5), 200 μM MV, 100 μl of cell suspension (with 0.02–0.04 mg of protein) and 10 mM KNO_3_ or 100 μM NaNO_2_ for MV-NR or MV-NIR activity respectively. Methyl viologen was reduced by the addition of freshly prepared sodium dithionite (dissolved in 300 mM NaHCO_3_ solution) at a final concentration of 14.4 mM.

### Haem-staining analysis

Aerobically grown *B. japonicum* cells were harvested by centrifugation, washed twice with BSN3 medium and resuspended in 500 ml of fresh medium of the same composition. Microaerobic conditions were then established with 2% (v/v) initial O_2_ concentration and cells were cultured for 48 h until a final *D* of ∼0.5 (at 600 nm) was reached. Cells were disrupted using a French pressure cell (SLM Aminco, Jessup) and membranes were isolated as described previously [26]. Membrane protein aliquots were diluted in sample buffer [124 mM Tris/HCl, pH 7.0, 20% (v/v) glycerol, 5% (v/v) SDS and 50 mM 2-mercaptoethanol] and incubated at room temperature for 10 min. Membrane proteins were separated at 4°C by SDS-PAGE [12% (w/v) acrylamide resolving gel with 20 μg of protein per lane], transferred to a nitrocellulose membrane and stained for haem-dependent peroxidase activity [38], using the chemiluminescence detection kit ‘SuperSignal’ (Pierce, Thermo Fisher Scientific). Protein concentration was estimated using the Bio-Rad assay (Bio-Rad Laboratories).

### Intracellular NO_2_^−^ determination

*B. japonicum* cells were harvested, washed and lysed by using a French pressure cell (SLM Aminco, Jessup). Soluble cell extracts were prepared by centrifugation at 10000 ***g*** for 30 min at 4°C and assayed for NO_2_^−^ using the method of Nicholas and Nason [39].

### NO consumption activity

NO consumption rates were determined using intact *B. japonicum* cells [obtained from BSN3 cultures with 2% (v/v) initial O_2_ and a *D* of ∼0.5 (at 600 nm)] with a 2 mm ISONOP NO electrode APOLLO 4000® (World Precision Institute). The reaction chamber (2 ml) was temperature-controlled, magnetically stirred and contained: 760 μl of 25 mM phosphate buffer (pH 7.4), 900 μl of cell suspension (4–5 mg protein), 100 μl of an enzyme mix containing *Aspergillus niger* glucose oxidase (40 units·ml^−1^) and bovine liver catalase (250 units·ml^−1^) (Sigma-Aldrich), 90 μl of 1 M sodium succinate and 100 μl of 320 mM glucose. Once a steady base line was obtained, 50 μl of a saturated NO solution (1.91 mM at 20°C) was added to the cuvette to start the reaction. Each assay was monitored until the NO detection had dropped to zero, i.e. when all NO was consumed.

### N_2_O measurements

*B. japonicum* cells were cultured as indicated above for NO consumption experiments, except that in addition to 2% (v/v) initial O_2_, the headspace of the cultures also contained 10% (v/v) acetylene in order to inhibit N_2_O reductase activity. After 96 h growth, gaseous samples were taken from the headspace of cultures. N_2_O was measured using an HP 4890D gas chromatography instrument equipped with an electron capture detector (ECD). The column was packed with Porapak Q 80/100 MESH and the carrier gas was N_2_ at a flow rate of 23 ml·min^−1^. The injector, column and detector temperatures were 125, 60 and 375°C respectively. The samples were injected manually using a Hamilton® Gastight syringe. Peaks corresponding to N_2_O were integrated using GC ChemStation Software (Agilent Technologies^©^) and the concentrations of N_2_O in each sample were calculated using N_2_O standards (Air Liquid).

## RESULTS

### Genetic basis for NO_3_^−^ and NO_2_^−^ assimilation in endosymbiotic denitrifying rhizobia

**Figure 1:**
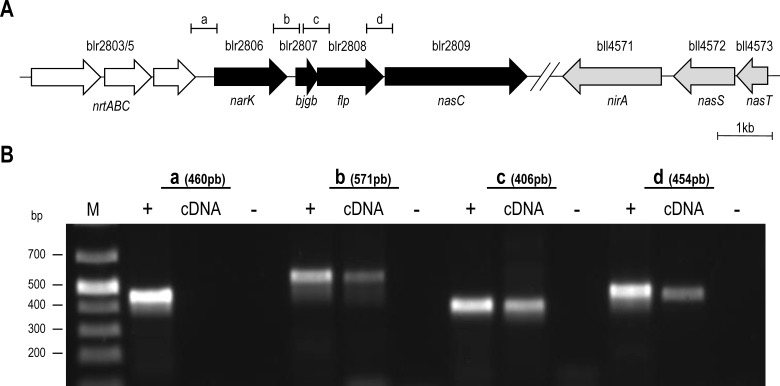
Organization of regulatory and structural genes for the assimilatory NO_3_^−^/NO_2_^−^ reductase pathway in *B. japonicum* (**A**) Schematic representation of the blr2803-5, blr2806-09 and bll4571-73 ORFs investigated in the present study. Putative intergenic regions probed by RT-PCR to determine the transcriptional architecture of the blr2806-09 region (i.e. *narK-bjgb-flp-nasC*) are labelled a–d. (**B**) The results for RT-PCR analysis obtained by agarose gel electrophoresis for regions a–d. Total RNA isolated from cells grown anaerobically with NO_3_^−^ served as the template for cDNA synthesis, whereas PCR amplifications using genomic DNA and without reverse transcriptase enzyme served as positive and negative controls respectively (as indicated above lanes).

**Figure 2:**
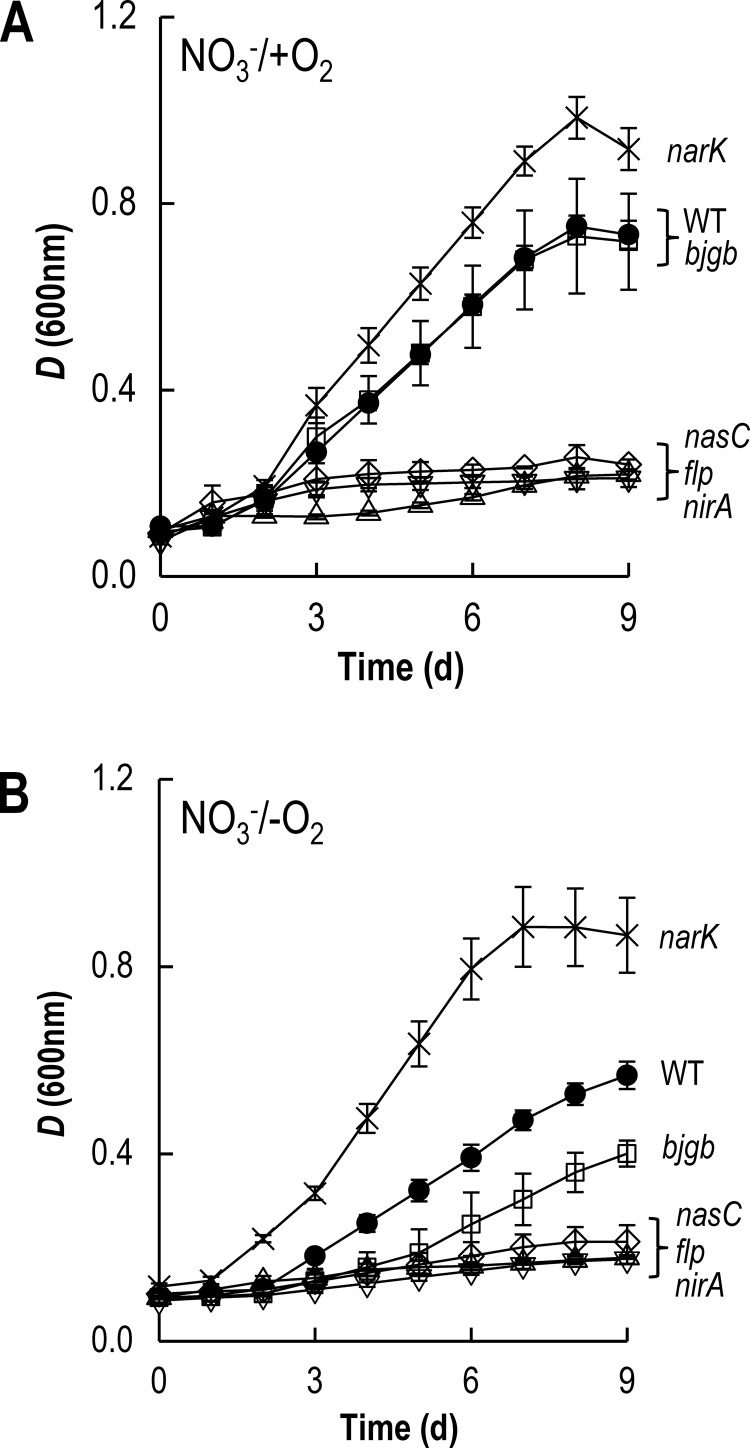
NO_3_^−^-dependent growth of *B. japonicum* Growth curves for WT (●), *narK* (×), *bjgb* (□), *flp* (▽), *nasC* (◇) and *nirA* (△) strains were measured under aerobic (**A**) and anaerobic (**B**) conditions in BN3 minimal medium with NO_3_^−^ as sole N-source. The results presented are the mean of two biological replicates assayed in triplicate.

*B. japonicum* USDA110 contains a putative assimilatory NO_3_^−^ reductase encoded at blr2809 (Figure 1A) [19,20]. Experiments confirmed that *B. japonicum* is able to grow aerobically or anaerobically using NO_3_^−^ as sole N-source with values for μ_max_ (app) (apparent maximum growth rate) of approximately 0.06 and 0.04 h^−1^ respectively (Figures 2A and 2B; Supplementary Table S3).

Notably, blr2809 lies downstream of several putative ORFs with predicted roles in N-metabolism (Figure 1A). To investigate the transcriptional architecture of this region, RT-PCR experiments were performed to detect intergenic regions (a–d). Here, specific cDNA was obtained for all regions except ‘a’ (Figure 1B). These findings reveal that blr2806–09 constitute a transcriptional unit. Thus, in-frame deletion strategies for subsequent molecular genetics experiments were adopted to prevent possible polar effects on co-transcribed genes (see ‘Experimental’ section for details).

**Figure 3:**
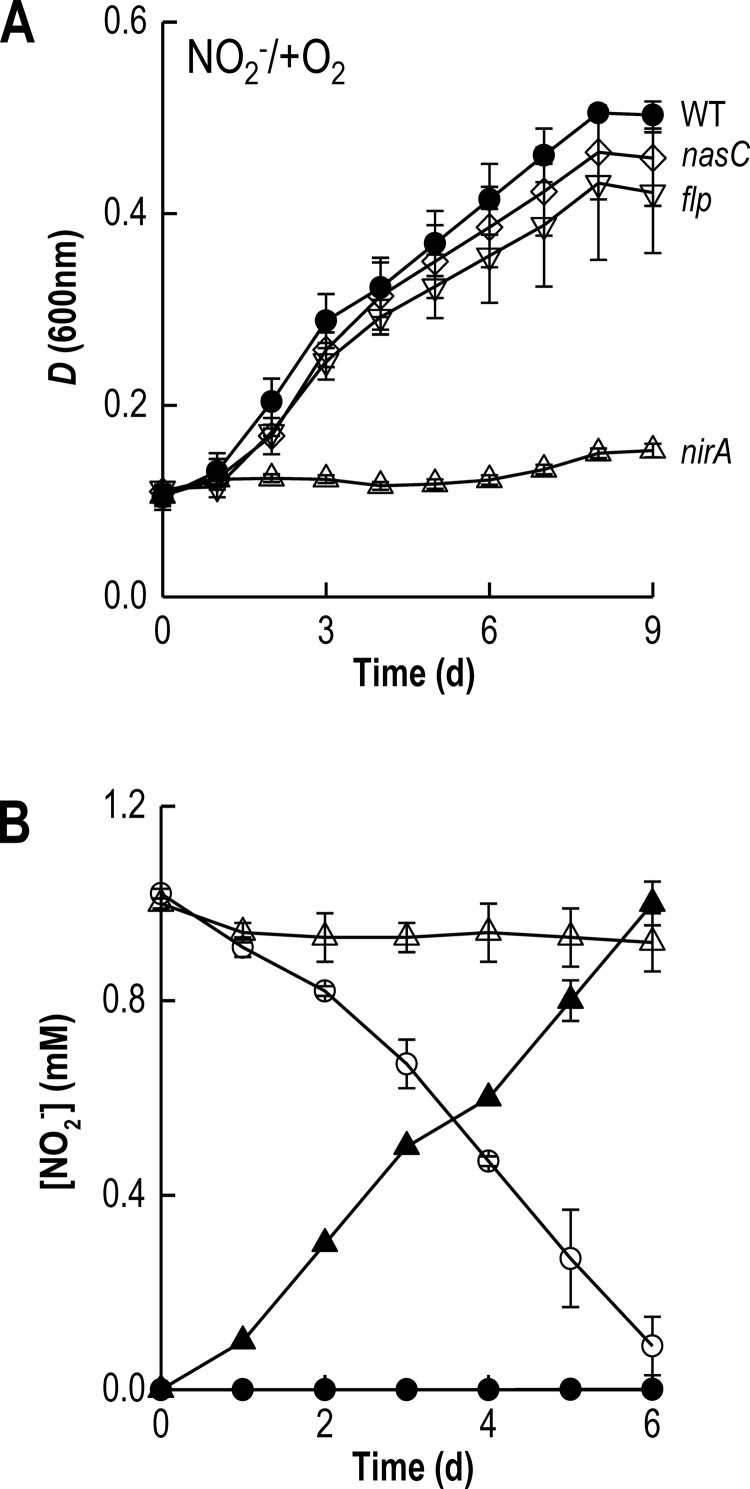
NO_2_^−^-dependent growth of *B. japonicum* (**A**) Growth curves for WT (●), *flp* (▽), *nasC* (◇) and *nirA* (△) strains were measured under aerobic conditions in BN2 minimal medium with NO_2_^−^ as sole N-source. (**B**) Extracellular NO_2_^−^ consumption (open symbols) and accumulation (solid symbols), using 1 mM NO_2_^−^ or 10 mM NO_3_^−^ as sole N-source respectively measured during growth of WT (circles) and *nirA* (triangles) strains. The results presented are the mean of two biological replicates assayed in triplicate.

Analysis of the amino acid sequence of blr2809 suggests the protein is a member of the molybdenum *bis*-molybdopterin dinucleotide cofactor binding superfamily and contains consensus motifs for co-ordination of an N-terminal [4Fe-4S] cluster and a C-terminal [2Fe-2S] cluster. This general organization is similar to other assimilatory NO_3_^−^ reductases, including *P. denitrificans* NasC and *Klebsiella oxytoca* NasA from the α- and γ-proteobacterial clades, respectively [25]. Accordingly, we adopt the α-proteobacterial nomenclature, NasC, for the *B. japonicum* protein encoded at blr2809 hereafter. A *B. japonicum* strain that was mutated by in-frame deletion of *nasC* lost the capacity for aerobic or anaerobic growth with NO_3_^−^ as sole N-source (Figures 2A and 2B; Supplementary Table S3). However, this strain retained the ability to grow using NO_2_^−^ as sole N-source and displayed similar growth kinetics to WT [μ_max_ (app) ∼ 0.03 h^−1^] (Figure 3A; Supplementary Table S3).

The genome of *B. japonicum* also contains an ORF for a putative assimilatory NO_2_^−^ reductase (*nirA*) at bll4571, a distinct locus situated ∼2 Mb from *nasC* on the chromosome (Figure 1A). NirA contains canonical cysteine-rich motifs in central and C-terminal sequence regions for iron–sulfur co-ordination and formation of the sirohaem NO_2_^−^ reductase/sulfite reductase ferredoxin half-domain respectively. However, NirA lacks N-terminal FAD- and NAD(P)H-binding domains present in bacterial NirB-type NAD(P)H-dependent NO_2_^−^ reductases [25]. Deletion of *nirA* resulted in *B. japonicum* being unable to grow aerobically or anaerobically with either NO_3_^−^ or NO_2_^−^ as sole N-source (Figures 2A, 2B and 3A; Supplementary Table S3). The ability of WT and *nirA* cells to consume 1 mM NO_2_^−^ during incubation experiments was tested (Figure 3B, open symbols). Whereas all NO_2_^−^ was removed from minimal medium after ∼6 days by WT cells, no significant decrease in extracellular NO_2_^−^ was observed in *nirA* mutant cultures (Figure 3B). Conversely, NO_2_^−^ production experiments using 10 mM NO_3_^−^, as sole N-source, revealed that WT cells did not accumulate NO_2_^−^ in the extracellular medium (Figure 3B, closed symbols). However, accumulation of ∼1 mM NO_2_^−^ was observed following incubation of the *nirA* mutant with NO_3_^−^ (Figure 3B). Thus, pre-cultured cells of the *nirA* mutant retained the capacity to reduce NO_3_^−^ to NO_2_^−^, but no further.

The putative flavoprotein (Flp), encoded at blr2808, contains canonical FAD- and NAD(P)H-binding domains typical of cytoplasmic NAD(P)H-dependent oxidoreductases present in several bacterial Nas operons [23] and is a strong candidate for mediating electron transfer to NasC and/or NirA. A *B. japonicum flp* mutant was unable to grow aerobically or anaerobically with NO_3_^−^ as the sole N-source (Figures 2A and 2B; Supplementary Table S3). However, the *flp* mutant displayed similar growth kinetics and yields [μ_max_ (app) ∼ 0.03 h^−1^, maximum *D* (at 600 nm)=0.43±0.08] to that observed for WT [μ_max_ (app) ∼ 0.03 h^−1^, maximum *D* (at 600 nm)=0.51±0.01] when cultured aerobically with NO_2_^−^ (Figure 3A; Supplementary Table S3). These findings suggest that Flp mediates electron transfer to NasC, but not to NirA. In order to confirm that deletion of *flp* did not influence expression of downstream genes, relevant strains were complemented with either pDB4017 (*nasC*) or pDB4015 (*flp*) constructs. The presence of pDB4017 and pDB4015 plasmids restored both aerobic and anaerobic growth of the *nasC* and *flp* mutants in the presence of NO_3_^−^ to near WT levels, thereby verifying the phenotypes observed (Supplementary Table S3).

Deletion of the blr2803–05 ORFs, predicted to encode an NrtABC-type NO_3_^−^ transporter, did not affect the capacity of the cells to grow with NO_3_^−^ as sole N-source (Supplementary Table S3). Bioinformatics analysis of blr2806 revealed that it encodes a putative member of the MFS of membrane proteins, sharing 66% and 59% amino acid similarity with the NO_3_^−^/NO_2_^−^ antiporters *E. coli* NarK [40] and *P. denitrificans* NarK2 [41] respectively (Supplementary Figure S1). Thus, we term this MFS-type transporter NarK rather than the generic ‘nitrite extrusion protein’ genome annotation currently assigned (http://genome.kazusa.or.jp/rhizobase/).

A *B. japonicum narK* mutant showed improved growth kinetics and yields when cultured aerobically [μ_max_ (app) ∼ 0.09 h^−1^, maximum *D* (at 600 nm)=0.98±0.05] or anaerobically [μ_max_ (app) ∼ 0.07 h^−1^, maximum *D* (at 600 nm) 0.89±0.09] with NO_3_^−^ as sole N-source when compared with aerobic [μ_max_ (app) ∼ 0.06 h^−1^, maximum *D* (at 600 nm)=0.73±0.12] or anaerobic [μ_max_ (app) ∼ 0.04 h^−1^, maximum *D* (at 600 nm)=0.61±0.03] growth of WT under the same conditions (Figures 2A and 2B; Supplementary Table S3). Furthermore, following 24 h aerobic growth, the *narK* mutant accumulated ∼2-fold higher levels of intracellular NO_2_^−^ than that accumulated by WT cells, i.e., 5.3±0.7 compared with 2.2±0.1 nmol NO_2_^−^ mg·protein^−1^ for the *narK* and WT strains respectively (Supplementary Figure S2). The addition of L-glutamate to minimal growth medium restored the inability of the *nasC*, *nirA* and *flp* mutants to grow with NO_3_^−^ under aerobic or anaerobic conditions (Supplementary Table S3). Under these conditions, growth yields obtained from the *narK* mutant were also similar to those obtained from WT cells (Supplementary Table S3). Collectively, these results confirm the importance of NarK, Flp, NasC and NirA for NO_3_^−^ assimilation by *B. japonicum*.

**Figure 4:**
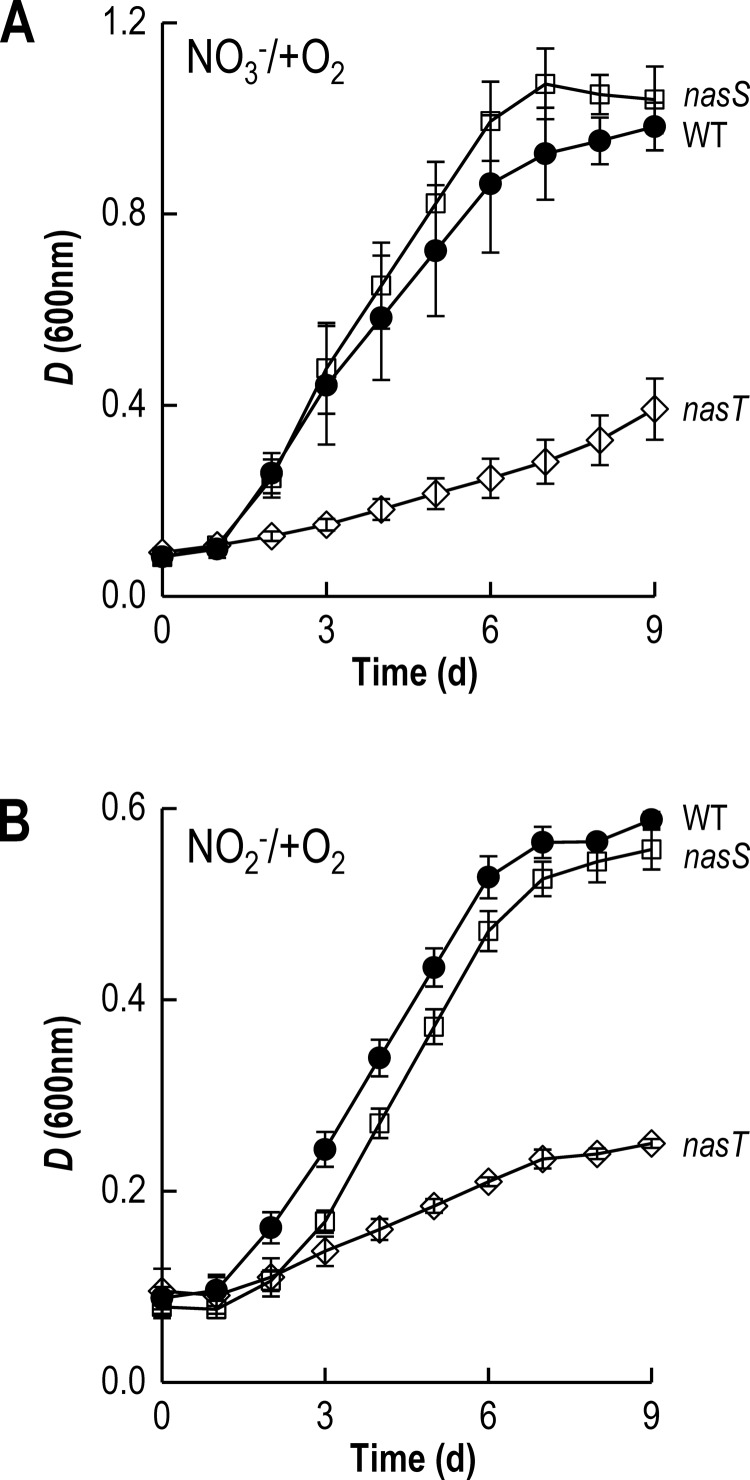
Growth curves for the *B. japonicum nasS* and *nasT* mutants Growth of WT (●), *nasS* (□) and *nasT* (◇) strains was measured in minimal medium, under aerobic conditions, with either NO_3_^−^ (**A**) or NO_2_^−^ (**B**), as sole N-source. The results presented are the mean of two biological replicates assayed in triplicate.

The regulatory proteins encoded by bll4573 (*nasT*) and bll4572 (*nasS*) constitute a NO_3_^−^/NO_2_^−^ responsive two-component system, NasS-NasT, which has been recently reported in *B. japonicum* [22]. A *B. japonicum nasT* mutant strain showed significant growth attenuation compared with the WT cells when cultured aerobically with either NO_3_^−^ (Figure 4A; Supplementary Table S3) or NO_2_^−^ (Figure 4B; Supplementary Table S3) as sole N-source, but growth of this strain was unaffected when cells were grown in the presence of L-glutamate (Supplementary Table S3). By contrast, a strain in which the *nasS* gene was mutated did not show a clear growth defect with respect to WT (Figure 4; Supplementary Table S3).

### A biochemical pathway for assimilation of NO_3_^−^ and NO_2_^−^

The biochemical basis of growth phenotypes observed for the various deletion strains was examined by enzymatic activity assay of whole cells, using dithionite-reduced methyl viologen, as an artificial electron donor. Here, MV-NR and MV-NIR activities were measured in WT and *nasC*, *nirA*, *flp*, *bjgb* and *narK* mutants, following aerobic incubation with NO_3_^−^ as sole N-source (Table 1). Since *B. japonicum* has periplasmic respiratory NO_3_^−^ reductase (NapABC) and NO_2_^−^ reductase (NirK) systems that might also use methyl viologen as an electron donor [26,42], control experiments using *napA* and *nirK* mutants were also performed in the present study. Importantly, and as expected, the respective MV-NR and MV-NIR activity levels observed in *napA* and *nirK* cells were similar to those observed in WT cells (Table 1), indicating that the contribution of the NapABC or NirK respiratory enzymes was not significant in cells cultured under aerobic conditions. This provided a solid platform for subsequent experiments.

**Table 1:** MV-NR and MV-NIR activities of *B. japonicum* strains incubated aerobically in minimal medium with NO**_3_**^−^ as sole N-source

		Activities	
*B. japonicum* str.	Genotype	MV-NR*	MV-NIR^†^
USDA 110	WT	32.0±5.2	6.9±0.9
GRPA1	*napA*	32.1±0.5	–
GRK308	*nirK*	–	7.5±0.8
4003	*nasC*	n.d.	7.2±1.2
4003-pDB4017	*nasC* (pDB4017)	30.5±4.8	–
4011	*nirA*	49.7±1.8	n.d.
4002	*flp*	68.3±6.7	6.1±0.8
4001	*bjgb*	28.4±5.0	6.1±0.4
4000	*narK*	35.8±2.2	10.9±1.5

*MV-NR and ^†^MV-NIR activities are expressed as nanomoles of NO_2_^−^ produced or consumed min^−1^·mg·protein^−1^. Data are expressed as the mean value±S.D. from at least two different cultures assayed in triplicate;–, not determined; n.d., not detectable.

Significantly, MV-NR activity was not detectable in *nasC* cells, but a similar level of MV-NIR activity was observed compared with WT cells. This was consistent with the loss of assimilatory NO_3_^−^ reductase expression, but not NO_2_^−^ reductase expression, in *nasC* cells (Table 1). MV-NR activity could be restored to WT levels in the *nasC* mutant, when the deletion was complemented with a corresponding plasmid-borne gene copy. Also, MV-NIR activity was absent from the *nirA* mutant (Table 1), consistent with the loss of assimilatory NO_2_^−^ reductase expression. However, the *nirA* mutant showed similar levels of MV-NR activity present in the parental strain following incubation with NO_3_^−^. Additional experiments revealed that MV-NR levels of *flp* cells showed an apparent ∼2-fold increase in activity compared with WT incubation with NO_3_^−^, but MV-NIR activity was relatively similar in both *flp* and WT cells (Table 1). That the absence of Flp (i.e. the proposed electron donor and partner to NasC) should increase MV-NR activity may result from modulation in catalytic activity of the isolated NasC protein. Alternatively, without Flp, the artificial chemical electron donor could have greater access to NasC and thus may enhance NO_3_^−^ reductase activity. Finally, as shown in Table 1, MV-NR and MV-NIR activities of *bjgb* or *narK* mutants were similar to those observed in WT cells.

### Regulation of the *narK-bjgb-flp-nasC* operon and *nirA* by NasS-NasT

In order to test the involvement of the NasT regulatory protein in NO_3_^−^-dependent induction of the *narK-bjgb-flp-nasC* operon and *nirA* gene, we examined expression of *narK-lacZ* and *nirA-lacZ* transcriptional fusion constructs in WT and *nasT* mutant cells following aerobic culture in the presence or absence of the inducer NO_3_^−^ (Table 2). Whereas similar low levels of β-galactosidase activity were observed from both fusions in WT cells incubated without NO_3_^−^, the presence of this molecule induced expression of the *narK-lacZ* and *nirA-lacZ* transcriptional fusions by approximately 5- and 3-fold respectively. However, β-galactosidase activity from the *narK-lacZ* reporter was undetectable in the *nasT* strain regardless of whether NO_3_^−^ was present or not (Table 2). Although similar basal levels of *nirA-lacZ* expression were observed in WT and *nasT* cells incubated without NO_3_^−^, a decrease of approximately 2-fold was found in *nasT* compared with WT when cells were incubated in the presence of NO_3_^−^ (Table 2).

**Table 2:** β-Galactosidase activity for *narK*-*lacZ* and *nirA*-*lacZ* fusions in *B. japonicum* WT, *nasS* or *nasT* strains Cells were cultured under aerobic conditions, in minimal medium, with or without NO_3_^−^ as sole N-source. Data are means±S.D. from at least three independent cultures, assayed in triplicate; n.d., not detectable.

		Miller units	
*B. japonicum* str.	Relevant genotype	–NO_3_^−^	+NO_3_^−^
4009	WT::*narK-lacZ*	153±40	759±54
4012-4009	*nasS*::*nark-lacZ*	972±132	897±66
4013-4009	*nasT*::*narK-lacZ*	n.d.	n.d.
4018	WT::*nirA-lacZ*	137±22	395±56
4012-4018	*nasS*::*nirA-lacZ*	412±37	372±31
4013-4018	*nasT::nirA-lacZ*	163±34	203±13

Additional studies to examine the role of NasS in NasT-dependent induction of the *narK-bjgb-flp-nasC* operon and *nirA* gene were also performed, using *narK-lacZ* or *nirA-lacZ* reporters. Here, β-galactosidase assays revealed that, in the absence of NO_3_^−^, the activity of each reporter fusion was significantly higher (approximately 6- and 3-fold for *narK-lacZ* and *nirA-lacZ* respectively) in *nasS* cells compared with WT cells (Table 2). These results imply that, in the absence of NO_3_^−^, NasS is a repressor of *narK-bjgb-flp-nasC* and *nirA* transcription. When equivalent experiments were performed in WT and *nasS* cells that had been pre-exposed to NO_3_^−^, expression levels for each reporter-fusion were very similar (Table 2).

Collectively, the reporter-fusion results suggest an inhibitory role for NasS in NasT-dependent induction of gene expression in *B. japonicum* and that NO_3_^−^-responsive control of both *narK-bjgb-flp-nasC* and *nirA* assimilatory gene expression is lost *in vivo* without NasS. This mode of regulation is analogous to NO_3_^−^/NO_2_^−^-responsive control of *nas* gene expression by NasS-NasT in the related α-proteobacterium *P. denitrificans* [21].

### Involvement of Bjgb and Flp in nitrosative stress defence

**Figure 5:**
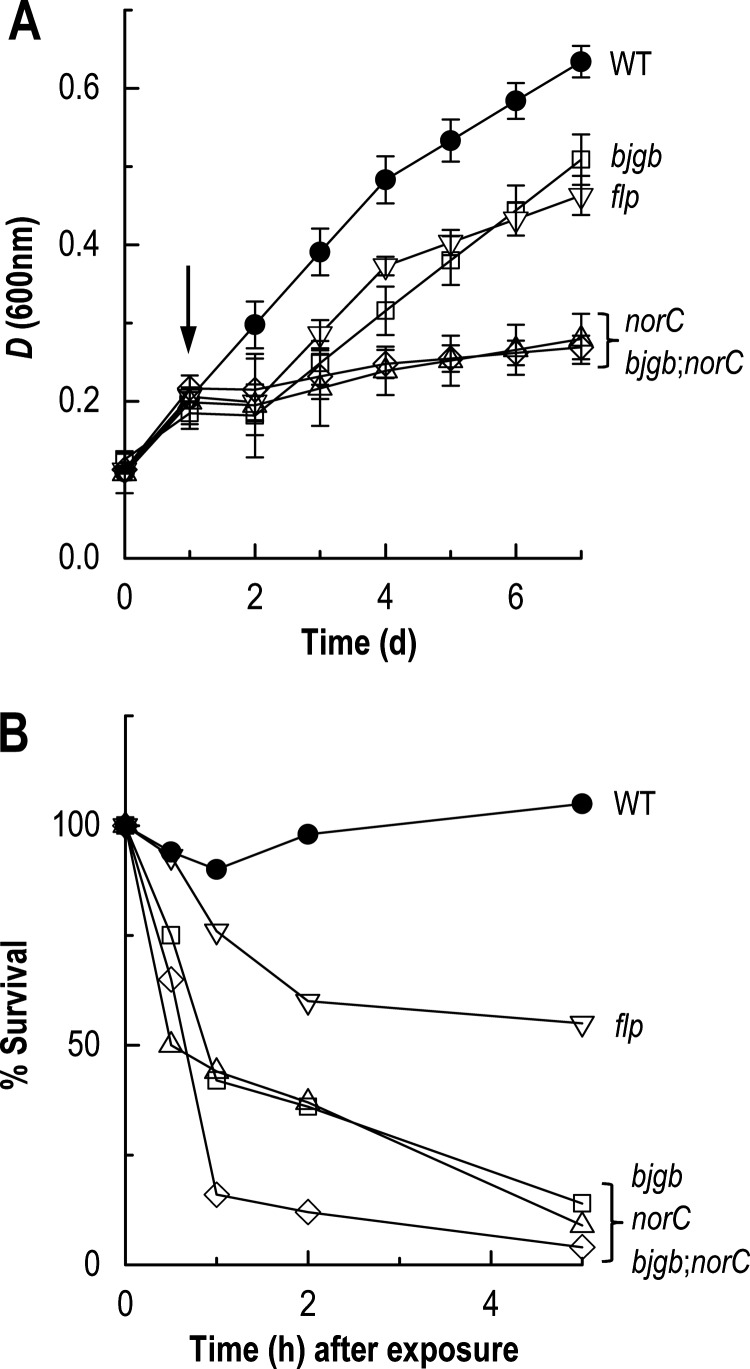
Growth inhibition curves (A) and cell viability assays (B) for *B. japonicum* WT (●), *bjgb* (□), *flp* (▽), *norC* (△) and *bjgb*;*norC* (◇) strains in response to nitrosative stress induced by addition of SNP For growth inhibition curves (**A**), *B. japonicum* strains were cultured microaerobically in BG minimal medium and 1 mM SNP was added after 1 d (as indicated by the arrow) and cell viability was measured from 1 to 5 h after exposure (**B**). The results presented are the mean of three biological replicates.

A marked difference in growth between the *bjgb* mutant [μ_max_ (app) ∼ 0.02 h^−1^, maximum *D* (at 600 nm)=0.45±0.02] and WT strains [μ_max_ (app) ∼ 0.04 h^−1^, maximum *D* (at 600 nm)=0.61±0.03] was observed under anaerobic conditions, in minimal medium with NO_3_^−^ as N-source (Figure 2B; Supplementary Table S3). By contrast, growth of the *bjgb* mutant and WT strains was similar under aerobic conditions (Figure 2A; Supplementary Table S3). These observations suggest that Bjgb has a key role *in vivo* for NO_3_^−^ assimilation under anaerobic conditions, but not during aerobic growth. Anaerobic NO_3_^−^ reduction is known to generate the potent cytotoxin NO, which requires NO-detoxification and nitrosative stress defence systems for bacterial survival [43,44]. To investigate the role of Bjgb in NO-metabolism, the nitrosative stress agent SNP was added (at 1 mM final concentration) to microaerobic *B. japonicum* cultures following growth in minimal medium with L-glutamate (BG) as sole N-source. Growth of WT cells was not significantly perturbed, whereas addition of SNP resulted in transient growth arrest of *bjgb* and *flp* strains that was restored after 24 h (Figure 5A). Perhaps most significantly, a *norC* or a *bjgb*;*norC* double mutant showed a substantially longer period of growth inhibition of approximately 7 days following addition of SNP to cultures (Figure 5A). The effect of SNP on cell viability was also assayed by performing viable cell counts on samples taken at intervals spanning a 5-h period following addition of SNP to cultures. Although WT cell viability was not significantly affected, addition of SNP caused a ∼60% decrease in cell survival for *norC* or *bjgb* cultures after 2 h (Figure 5B). The most prominent effect was observed with the *bjgb*;*norC* double mutant, which was the most sensitive to nitrosative stress. Here, approximately 80% of cells were killed within 1–2 h following SNP exposure (Figure 5B). Furthermore, the addition of SNP provoked a ∼40% decrease in *flp* viability after 2 h incubation. These results revealed the importance of Bjgb and Flp for protection against nitrosative stress in *B. japonicum* under free-living conditions. NO is a product of SNP breakdown and a similar sensitivity of *bjgb* or *flp* mutants to NO was observed using spermine NONOate as an NO-generating compound (result not shown). Importantly, complementation with pDB4014 (harbouring a functional plasmid-borne copy of *bjgb*) allowed the *bjgb* mutant to grow anaerobically with NO_3_^−^ to near WT levels (Supplementary Table S3). This confirmed that the growth phenotype observed for the *bjgb* mutant was not caused by a downstream effect on *flp* gene expression.

### NO formed during NO_3_^−^ assimilation induces *nor* gene expression

To further investigate the role of Bjgb and Flp in NO metabolism, the ability of *B. japonicum bjgb* and *flp* strains to consume NO was analysed. Here, cells were incubated in BSN3 medium, with 2% initial O_2_ and NO consumption rates were determined using an NO-electrode (Supplementary Figure S3). A ∼2.5-fold increase in NO consumption was observed in the *bjgb* mutant compared with the WT strain (Table 3). This increase was not observed in the *flp* mutant, which showed NO consumption rates marginally lower than that observed in WT cells (Table 3; Supplementary Figure S3). NO consumption in the *norC* or the *bjgb*;*norC* mutants was approximately 1.6- and 1.7-fold lower respectively, compared with that observed in WT cells (Table 3; Supplementary Figure S3). The presence of residual activity in the *bjgb*;*norC* implies that under our experimental conditions, another enzyme(s) or perhaps a chemical process may be involved in NO consumption. The ability of *bjgb* cells to produce N_2_O following incubation in BSN3 medium with 2% initial O_2_ was also investigated. The *bjgb* mutant produced approximately 2.5-fold more N_2_O than WT cells. By contrast, the level of N_2_O produced by the *flp* mutant was comparable to WT (Table 3). Given that N_2_O production was not detected for either the *norC* or the *bjgb*;*norC* mutants, this suggested the NorCB enzyme was the main source of N_2_O *in vivo*.

**Table 3:** NO consumption activity and N_2_O levels for *B. japonicum* WT, *bjgb*, *flp*, *norC* and *bjgb*;*norC* strains cultured in BSN3 minimal medium under 2% (v/v) initial O_2_ Data are expressed as the means±S.D. from at least two different cultures assayed in triplicate; n.d., not detectable.

*B. japonicum* str.	Genotype	NO consumption activity (nmol·h^−1^·mg·protein^−1^)	N_2_O (mM)
USDA110	WT	155±29	1.04±0.26
4001	*bjgb*	384±65	2.34±0.16
4002	*flp*	101±17	0.88±0.03
GRC131	*norC*	97±14	n.d.
GRC131-4001	*bjgb*;*norC*	92±18	n.d.

**Figure 6:**
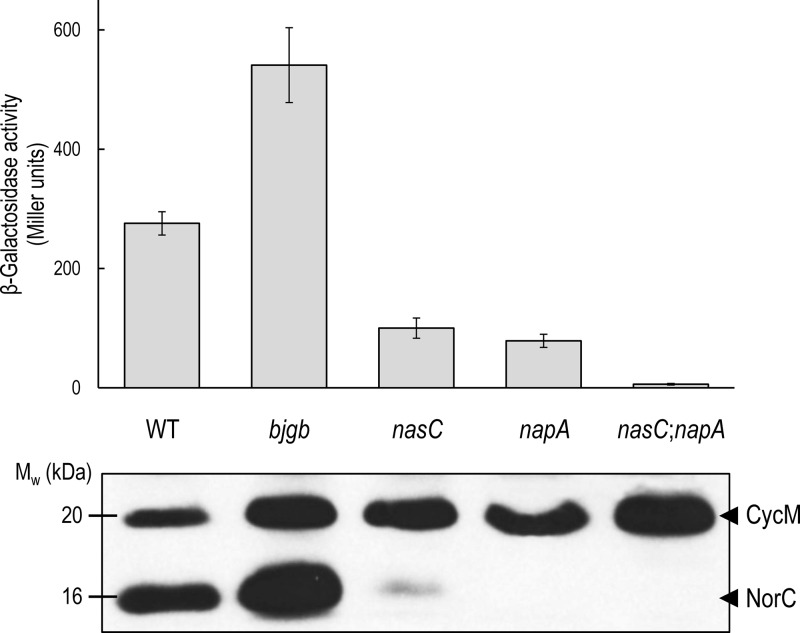
Expression of *B. japonicum nor* genes during NO_3_^−^-dependent growth β-Galactosidase expression levels for the *norC-lacZ* transcriptional fusion in the WT, *bjgb*, *nasC*, *napA* and *nasC*;*napA* strains grown in BSN3 minimal medium containing 2% initial O_2_ (v/v) and NO_3_^−^ as sole N-source. Haem-staining SDS-PAGE analysis of membrane fractions from *B. japonicum* strains is inset below. Each lane contains ∼20 μg of total protein for the strains described. Haem-staining bands for previously identified *c*-type cytochromes, CycM and NorC, are indicated.

To test whether the higher levels of NO consumption and N_2_O production observed by the *bjgb* mutant were due to an induction of NorCB expression, *norC* transcription and relative abundance of NorC in membrane extracts were analysed, using a *norC-lacZ* transcriptional fusion and haem staining SDS-PAGE respectively. Firstly, a ∼2-fold increase in *norC-lacZ* expression was observed in the *bjgb* mutant compared with WT (Figure 6). Given that the *norC* promoter is highly sensitive to N-oxides, including NO [31], an induction of β-galactosidase activity implies that Bjgb may act as a net sink for NO in WT cells. By contrast, β-galactosidase activity of the *norC-lacZ* transcriptional fusion was similar for both the *nasC* and the *napA* mutants, being approximately 3-fold lower compared with WT levels (Figure 6). Activity of the *norC-lacZ* transcriptional fusion was essentially abolished in the *nasC*;*napA* double mutant, implying that NO_3_^−^ reduction by NasC or NapA was the source of NO required for *norC-lacZ* expression (Figure 6).

SDS-PAGE analysis of membranes (that were normalized for total protein) by haem staining was used as a qualitative assay for expression of the NorC cytochrome. In *bjgb* cells, NorC levels were significantly increased relative to WT (Figure 6 inset; compare lanes 1 and 2). However, a clear decrease in NorC expression was observed in the *nasC* mutant compared with WT (Figure 6 inset; compare lanes 1 and 3). Furthermore, haem staining failed to detect NorC expression in membranes prepared from either the *napA* or the *nasC*;*napA* mutant (Figure 6 inset; compare lane 1 with lane 4 or 5).

## DISCUSSION

### Defining the key components and transcriptional architecture of NO_3_^−^ and NO_2_^−^ assimilation in *B. japoniucum*

A series of molecular genetics studies have established that genes encoded at two distinct loci, blr2806–09 and bll4571–73 of the *B. japonicum* genome (http://genome.kazusa.or.jp/rhizobase/), encode structural and regulatory components of a combined assimilatory NO_3_^−^ reductase and NO detoxification system (Figure 1). RT-PCR experiments demonstrate that the *narK-bjgb-flp-nasC* genes (present at blr2806–09 respectively) constitute a transcriptional unit. However, three putative genes (blr2803–05) predicted to encode a NO_3_^−^ transport system (similar to NrtABC, reviewed in [45]) and that lie immediately upstream of the *narK* operon are transcribed from a different promoter. The *nasTS-nirA* gene cluster (present at bll4571–73 respectively) lies some 2 Mb from the *narK* operon in the genome and encodes a NO_3_^−^/NO_2_^−^ responsive two-component regulatory system, NasS-NasT [22] and a putative ferredoxin-dependent NO_2_^−^ reductase (NirA).

A role for the *bjgb* (blr2807) gene product in NO detoxification has been described [3,20], but the functions of other putative proteins encoded within the *narK* operon and biochemical components for the assimilatory NO_3_^−^ reductase pathway in *B. japonicum* were unknown. In the present work, we have demonstrated that the assimilatory NO_3_^−^ reductase (we rename herein as NasC) is encoded by blr2809 and is essential for NO_3_^−^-dependent growth. The second core cytoplasmic enzyme component of the NO_3_^−^ assimilation pathway is NirA, which is required for growth on either NO_3_^−^ or NO_2_^−^ as sole N-source. Consistent with our findings, it has recently been demonstrated that NirA (encoded by bll4571) is required for utilization of NO_3_^−^ or NO_2_^−^ as sole N-source in *B. japonicum* [46]. NO_3_^−^-dependent induction of *nasC* (as part of the *narK* operon) and *nirA* expression is mediated by the two-component regulator NasS-NasT, an observation that is consistent with the role of this system in other α-proteobacteria [21].

**Figure 7:**
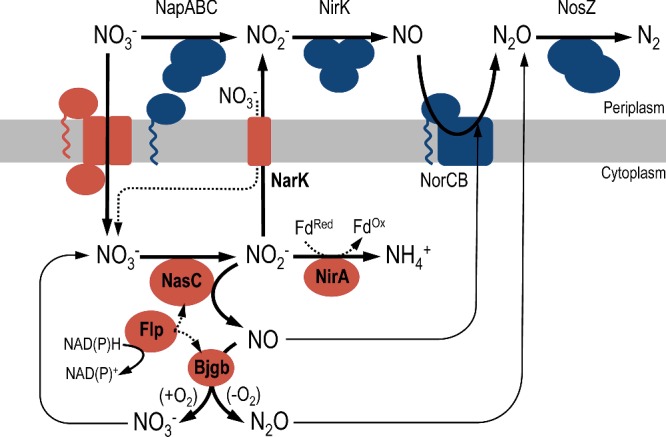
Proposed biochemical pathway for NO_3_^−^-assimilation and NO-detoxification (red), alongside well-characterized systems for dissimilatory NO_3_^−^ respiration (blue) in *B. japonicum* Assimilatory reduction of NO_3_^−^ to NH_4_^+^ is performed by sequential action of the NO_3_^−^ reductase NasC and ferredoxin (Fd)-dependent NO_2_^−^ reductase NirA. Electrons from NAD(P)H are supplied to NasC and also Bjgb by Flp. During assimilatory NO_3_^−^ reduction, cytoplasmic NO_2_^−^ may accumulate and be further reduced, by NasC, to generate cytotoxic NO. NarK can counteract accumulation of NO_2_^−^ by exporting it to the periplasm. Alternatively, Bjgb may detoxify the NO, formed by adventitious reduction in cytosolic NO_2_^−^, to NO_3_^−^ or N_2_O in the presence or absence of O_2_ respectively. Expression of NorCB is up-regulated during NO_3_^−^ assimilation and this respiratory system may assist Bjgb to limit accumulation of NO and maintain cell viability.

Phenotypic analyses of a mutant lacking Flp (encoded by blr2808) suggest that Flp mediates electron transfer to NasC, but not to NirA. Consecutive genes from the same operon encode Flp and NasC, but lie in a different genetic locus to bll4571 (*nirA*). This genetic organization may explain the requirement of Flp for NO_3_^−^ assimilation but not for NO_2_^−^ assimilation, which instead is ferredoxin dependent (Figure 7). In contrast with *B. japonicum*, in *P. denitrificans* the regulatory and structural elements for a cytoplasmic NO_3_^−^/NO_2_^−^ reductase system comprise a large gene cluster, *nasTSABGHC* [23]. The absence of a *nasG* homologue in either the *narK* operon or the *nirA* cluster in *B. japonicum* is notable. NasG may mediate electron flux to both the NO_3_^−^ reductase and the NO_2_^−^ reductase in other bacteria to prevent accumulation of excess NO_2_^−^ by NO_3_^−^ reduction in the cytoplasmic compartment [23,25]. Instead, for *B. japonicum*, genes encoding systems for NO_2_^−^ transport and NO-detoxification are present within the operon encoding the NO_3_^−^ reductase, which generates NO_2_^−^.

Sequence comparison of blr2806 with homologous proteins from diverse bacterial phyla suggests that this gene encodes an MFS-type NO_3_^−^/NO_2_^−^ antiporter with similarity to *E. coli* NarK. The capacity of a *B. japonicum narK* mutant to accumulate NO_2_^−^ inside the cell demonstrates the involvement of NarK in NO_2_^−^ export. Further, phenotypic analyses reveal that NarK is not the main system for cytoplasmic NO_3_^−^ import, as *narK* cells were still able to grow on NO_3_^−^. Instead, the *narK* mutant showed enhanced growth compared to WT cells with NO_3_^−^ as sole N-source, either under aerobic or anaerobic conditions. These observations imply that NarK acts to lower cytoplasmic NO_2_^−^ levels by exporting NO_2_^−^ to the periplasm and this process may involve corresponding import of NO_3_^−^ (Figure 7) [40]. In this respect, it is significant that *B. japonicum* NarK performs a very different role to the MFS-type NO_3_^−^/NO_2_^−^ transporter NasA, which supplies NO_3_^−^ to the cytoplasmic NO_3_^−^/NO_2_^−^ reductase pathway in other α-proteobacteria [23]. Instead, by counteracting NO_2_^−^ accumulation, the *B. japonicum* NarK protein may thus represent a first level of protection to mitigate the production of cytotoxic NO, by adventitious reduction of NO_2_^−^ within the cytoplasm [43]. However, as a consequence, in WT cells NarK may also lower substrate availability for NirA and thus limit growth on NO_3_^−^.

Deletion of blr2803–05 that bioinformatics analyses had predicted to collectively encode an NrtABC family transporter, did not affect the ability of *B. japonicum* to assimilate NO_3_^−^ as sole N-source. Therefore, the main route(s) for assimilatory NO_3_^−^ import remains to be established. Although blr2803–05 are not required for NO_3_^−^ assimilation, there are other NtrABC-like candidates present on the chromosome (e.g. bll5732–34) that may facilitate NO_3_^−^ import to the cytoplasm.

### A modular detoxification system for NO generated during NO_3_^−^ assimilation

In general, Nas systems have a high degree of structural plasticity, yet most contain proteins for transport and reduction of NO_3_^−^ and NO_2_^−^ [23,25,45,47,48]. In the present work, a novel NO_3_^−^ assimilation system that also includes proteins for NO-detoxification is reported. The *narK-bjgb-flp-nasC* operon in *B. japonicum* encodes the sdHb Bjgb [3,20], which is homologous to the N-terminal haem-containing domain of *E. coli* FHb (Hmp) as well as the sdHbs from *Vitreoscilla stercoraria* (Vgb) and *Campilobacter jejuni* (Cgb) [20].

Deletion of *bjgb* had a strong negative affect on O_2_-limited growth with NO_3_^−^ as sole N-source, relative to WT, which implies a role for Bjgb in protecting *B. japonicum* cells from nitrosative stress. Importantly, in the absence of Bjgb, NO_3_^−^ respiring cells were also highly sensitive to exogenous NO. Since growth of the *bjgb* mutant was not affected under aerobic conditions, the role of Bjgb may be restricted to anaerobic NO_3_^−^-dependent growth. However, our data suggest that the contribution of Bjgb to N_2_O production *in vivo* is low. These observations are consistent with studies performed in *E. coli*, which reveal Hmp can reduce NO to N_2_O under anaerobic conditions, but with a much lower rate compared with the activity of the FlRd NorV [44]. Furthermore, expression of the respiratory NorCB is significantly up-regulated in the *bjgb* mutant, relative to WT (see Figure 6), in response to increased intracellular NO levels that arise during NO_3_^−^-dependent growth. This result suggests that increased NorCB expression may counteract accumulation of cytotoxic NO and may partially compensate for the absence of the cytoplasmic Bjgb NO-detoxification system to maintain cell viability, albeit with a detrimental impact on anaerobic growth. Consequently, the bulk of the N_2_O produced by the *bjgb* mutant can be attributed to NorCB activity, which is increased by ∼2-fold relative to WT levels.

In *E. coli* Hmp, the FAD prosthetic group within the C-terminal NADH-reductase domain provides electrons from NAD(P)H that are required to reduce the NO-bound haem active site and complete the catalytic cycle. Aside from NO dioxygenation, Hmp has also been shown to perform slower reduction of NO to N_2_O under anoxic conditions, which operates at approximately 1% of the rate observed for aerobic dioxygenase activity [49–52]. In the case of Cgb (an sdHb family protein that like Bjgb lacks the reductase domain present in the FHb Hmp), the electron–donor protein remains to be identified. However, recent heterologous expression studies of Cgb in *E. coli* have reported a minor role for the NADH-(flavo)rubredoxin oxidoreductase NorW [53]. In *B. japonicum*, the enhanced sensitivity of the *flp* mutant to chemical NO-donors suggests that Flp may supply electrons from NAD(P)H that are required for Bjgb activity (Figure 7).

### Sources of NO: NasC and NapA activity is responsible for elevated NorCB expression

In eukaryotes, NO synthase (NOS) enzymes have been well described as the main NO-forming pathway for cell signalling and anti-microbial host defence [54]. By contrast, NO-formation in prokaryotes has been considered a by-product of denitrification, anaerobic ammonium oxidation and other related respiratory pathways [55–58]. However, NO is now increasingly recognized as a key substrate for ‘non-respiratory’ pathways in bacteria, e.g. those that protect against nitrosative stress and the link between NO-detoxification and pathogenicity has been the focus of several studies (reviewed by Maia and Moura [56,59]). The biochemical basis for NO-formation during anaerobic bacterial respiration has been shown to result from enzymic reduction of the pseudo-substrate NO_2_^−^ by the respiratory membrane-bound NO_3_^−^ reductase, Nar [43,60,61]. Furthermore, a small contribution (less than 3%) has been attributed to the periplasmic enzyme, Nap [43,61]. In the context of this present study, the potential contribution of cytoplasmic NO_2_^−^ reduction to NO formation, by NasC, during NO_3_^−^/NO_2_^−^ assimilation has not yet been investigated.

In the denitrifying endosymbiotic bacterium *B. japonicum*, reduction of NO_2_^−^ by the periplasmic copper-dependent NO_2_^−^ reductase NirK is the main NO-forming process, which occurs during anaerobic NO_3_^−^ respiration [1,42]. Many studies have proposed that NO activates transcription of *nor* genes and that this control is mediated by regulatory proteins designated NNR/NnrR and DNR (reviewed by Spiro [18,62]). In the present study, we demonstrate that cells lacking the periplasmic respiratory NO_3_^−^ reductase NapA, where NO synthesis from denitrification is blocked, results in very low expression of NorCB. Perhaps our most important finding was that, in addition to NapA, the assimilatory NO_3_^−^ reductase (NasC) is also responsible for generating NO, as induction of NorCB was significantly lowered and completely abolished, relative to WT, in the *nasC* and *nasC*;*napA* mutant strains respectively (Figure 6). Therefore, the importance of NasC not only in NO_3_^−^ assimilation but also in NO production has been demonstrated.

Co-expression of *bjgb*, *flp* and *nasC* that constitute a combined NO_3_^−^ assimilation/NO-detoxification system may represent a novel method by which bacteria maintain cytoplasmic NO homeostasis and protect against nitrosative stress imposed during NO_3_^−^-dependent growth, where pathways for both respiratory denitrification and NO_3_^−^/NO_2_^−^ assimilation are active (Figure 7). Although co-regulation between similar NO-forming and consuming systems has been proposed in *Aspergillus nidulans* [63], to our knowledge, this is the first time where this mechanism has been reported in bacteria. Finally, should production of NO exceed concentrations that can be contained by Bjgb-Flp, a ‘safety’ mechanism exists to enhance expression of NorCB to drive reduction of excess NO to N_2_O.

## Abbreviations

Bjgb: *Bradyrhizobium japonicum* haemoglobin
FHb: flavohaemoglobin
Flp: flavoprotein
FlRd: flavorubredoxin
MFS: major facilitator superfamily
MV-NIR: methyl viologen-dependent nitrite reductase
MV-NR: methyl viologen-dependent nitrate reductase
NapABC: periplasmic respiratory quinol/nitrate oxidoreductase
NarK: nitrate/nitrite transporter
Nas: assimilatory nitrate reductase
NirA: assimilatory nitrite reductase
NirK: copper-dependent respiratory nitrite reductase
NorCB: cytochrome-*c* nitric oxide reductase
NosZ: nitrous oxide reductase
PSY: peptone-salts-yeast extract
sdHb: single-domain haemoglobin
SNP: sodium nitroprusside
WT: wild-type
μ_max_ (app): apparent maximum growth rate
